# Exploring the adoption of less restricted criteria for respiratory syncytial virus prophylaxis in late preterm infants: insights from a retrospective analysis

**DOI:** 10.3389/fped.2023.1154518

**Published:** 2023-06-08

**Authors:** Vito Mondì, Piermichele Paolillo, Manuela Bedetta, Natalia Lucangeli, Simonetta Picone

**Affiliations:** Neonatology and Neonatal Intensive Care Unit, Policlinico Casilino Hospital, Rome, Italy

**Keywords:** respiratory syncytial virus, bronchiolitis, prophylaxis, late preterm, risk factor

## Abstract

**Background:**

Preterm infants born between 33 and 35 weeks of gestational age (wGA) have been considered a “major underserved population” and ineligible to receive palivizumab (PLV), the only drug authorized to date for respiratory syncytial virus (RSV) prophylaxis, by current international guidelines. In Italy, such a vulnerable population is currently eligible for prophylaxis, and, in our region, specific risk factors are taken into consideration (SIN_Lazio_ score) to target prophylaxis for those at highest risk. Whether the adoption of less or more restrictive eligibility criteria for PLV prophylaxis would translate into differences in bronchiolitis and hospitalization incidence is not known.

**Materials and methods:**

A retrospective analysis was conducted in 296 moderate-to-late preterm infants (born between 33 and 35^+6^ weeks) who were being considered for prophylaxis in two epidemic seasons: 2018–2019 and 2019–2020. The study participants were categorized according to both the SIN_Lazio_ score and the Blanken risk scoring tool (BRST), which was found to reliably predict RSV-associated hospitalization in preterm infants on the basis of three risk factor variables.

**Results:**

Based on the SIN_Lazio_ score, approximately 40% of infants (123/296) would meet the criteria to be eligible for PLV prophylaxis. In contrast, none of the analyzed infants would be considered eligible for RSV prophylaxis on the basis of the BRST. A total of 45 (15.2%) bronchiolitis diagnoses were recorded on average at 5 months of age in the overall population. Almost seven out of 10 (84/123) patients exhibiting ≥3 risk factors to be eligible for RSV prophylaxis according to SIN_Lazio_ criteria would not be receiving PLV if they were categorized on the basis of the BRST. Bronchiolitis occurrence in patients with a SIN_Lazio_ score ≥3 was approximately 2.2 times more likely than that in patients with a SIN_Lazio_ score <3. PLV prophylaxis has been associated with a 91% lower risk of requiring a nasal cannula.

**Conclusion:**

Our work further supports the need for targeting late preterm infants for RSV prophylaxis and calls for an appraisal of the current eligibility criteria for PLV treatment. Therefore, adopting less restrictive criteria may ensure a comprehensive prophylaxis of the eligible subjects, thus sparing them from avoidable short- and long-term consequences of RSV infection.

## 1. Introduction

Globally, respiratory syncytial virus (RSV) contributes substantially to the burden of morbidity and mortality in children of ≤5 years of age, with the occurrence of over 30 million RSV-associated acute lower respiratory tract infection (LTRI) episodes, one-tenth of which leads to hospitalization (1). In infants (0–12 months of age), a higher risk of RSV-associated hospitalization has been reported, with almost one in five infants experiencing RSV-associated LTRI being admitted to hospital (1–3). Infants who experience RSV infection in the first months of life often require intensive care unit (ICU) admission and are at risk of developing wheezing and asthma (4). Therefore, aside from the currently available therapeutic measures that are mostly supportive, preventing RSV infection is relevant to reduce both the associated morbidity and the substantial economic burden of the long-term complications of RSV disease (5). Palivizumab (PLV) is the only licensed immuno-prophylaxis available to prevent severe RSV LTRI in specific high-risk pediatric populations. In Italy, reimbursement criteria for PLV include the following: infants born at 29 weeks of gestational age (wGA) or less and less than 12 months of age, infants born at 35 wGA or less and less than 6 months of age, children less than 2 years of age and requiring treatment for bronchopulmonary dysplasia (BPD) within the last 6 months, and children less than 2 years of age and with hemodynamically significant congenital heart disease at the onset of the RSV season (6).

Infants born at 34–36 completed gestational weeks account for most preterm infants: overall, in Italy, premature babies are equal to 6.3% of the total newborns in 2020; in detail, 0.7% 32–33 wGA, 4.8% 34–36 wGA (7). Compared with full term infants (≥37 wGA), late preterm (34–36 wGA) infants exhibit higher neonatal morbidity and mortality with an increased healthcare burden (8). Mounting evidence suggests that infants born between 33 and 35 wGA have a higher risk of hospitalization due to RSV infections than full-term infants (9, 10), but comparable to that seen in very preterm infants (11) with a greater ICU and hospital length of stay and higher rates of medical complications, intubation rates, and healthcare costs than infants in any other gestational age group (12). Since moderate-to-late preterm infants (33–36 wGA) constitute most of the preterm births on neonatal care, they should not be disregarded when RSV prevention interventions are implemented (8). Interrupted lung development and an immature immune system have been linked to an increased susceptibility to RSV LRTI, along with other environmental, social, and physiological risk factors. In addition, preterm infants have deficiencies in both innate and adaptive immunity, and in the interaction between these two systems (13, 14). However, despite being such a vulnerable population, the late preterm was not included in the American Academy of Pediatrics (AAP) 2014 guidelines for prophylaxis with PLV (15). Of note, when, in line with AAP guidelines, the Italian Drug Agency (AIFA) modified the indication for financial coverage in 2016 (16) by limiting it to infants with ≤29 wGA and infants with BPD born <32 wGA, a rising trend in rates of bronchiolitis and bronchiolitis-related hospitalization has been reported by several Italian hospitals, including our unit (17). When the reimbursement restrictions for infants born at GA 30– ≤35 weeks were subsequently removed by AIFA in late 2016, the proportion of infants experiencing bronchiolitis declined significantly (26% vs. 10.7%, *p* = 0.048), as all eligible infants could receive PLV prophylaxis (18).

To effectively tackle RSV burden, it is desirable to identify the vulnerable set of infants at risk of severe infection and eligible for intervention, thus sparing them an avoidable bronchiolitis and potential hospitalization. However, a big debate as to which type of patients should receive RSV prophylaxis is ongoing, with heterogeneous recommendations being made for PLV prophylaxis among countries and a mounting body of evidence exploring the appropriateness of prophylaxis in preterm infants of ≥29 wGA (19, 20). The use of risk factors may provide a pragmatic approach to targeting prophylaxis for preterm infants at highest risk. Recently, a scoring tool [the Blanken Risk Scoring Tool (BRST)] was proposed to reliably predict the risk of RSV-associated hospitalization in moderate-late preterm infants (32–35 wGA), on the basis of three risk factor variables (21). However, such a tool has been developed on the basis of a dataset prepared from six individual studies with different objectives and design, which influenced the included gestational age ranges of infants and how and what risk factors were collected. In Italy, prophylaxis with PLV is supervised by the Italian Neonatology Society guidelines (22), which allows each region to tailor national recommendations to specific local needs. In the Lazio region, PLV prophylaxis is recommended to be started on November 1st in infants with 33–35 wGA and aged <6 months at the beginning of the epidemic RSV season, with at least, or more than, three of the following risk factors: male gender; smoking exposure; surfactant therapy; siblings <10 years old; living in crowded conditions and/or in unhealthy households and early childcare (23). Our hospital ranks first in the Lazio region, with 4,294 deliveries recorded in 2020 (24, 25).

The aim of our retrospective analysis was to investigate whether the adoption of less or more restricted eligibility criteria for PLV prophylaxis would translate into differences in bronchiolitis and hospitalization incidence in late preterm infants (born between 33 and 35^+6^ wGA) who have not been found eligible for prophylaxis so far by international guidelines.

## 2. Materials and methods

**Figure 1:**
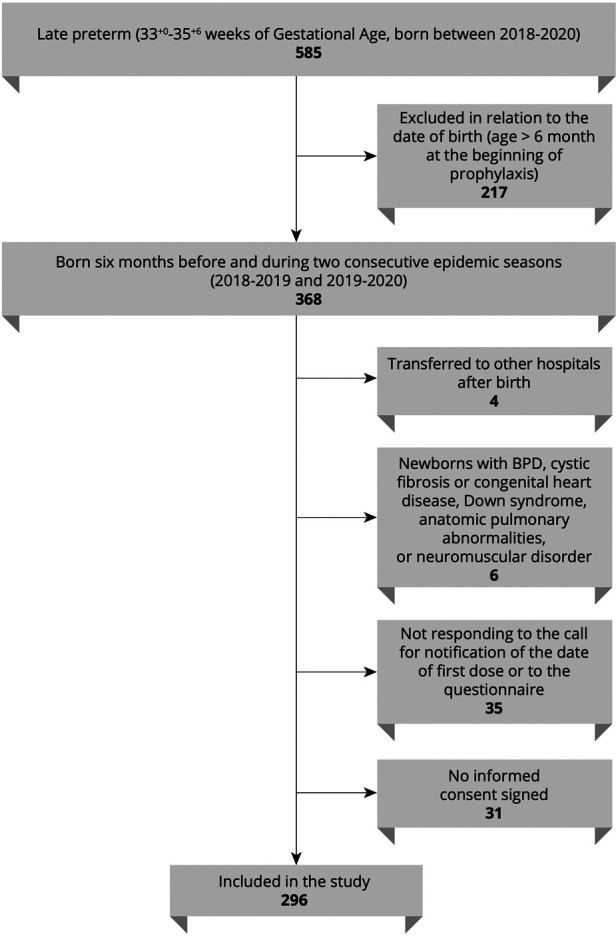
Flowchart of the participants in the study. BPD, bronchopulmonary dysplasia.

**Figure 2:**
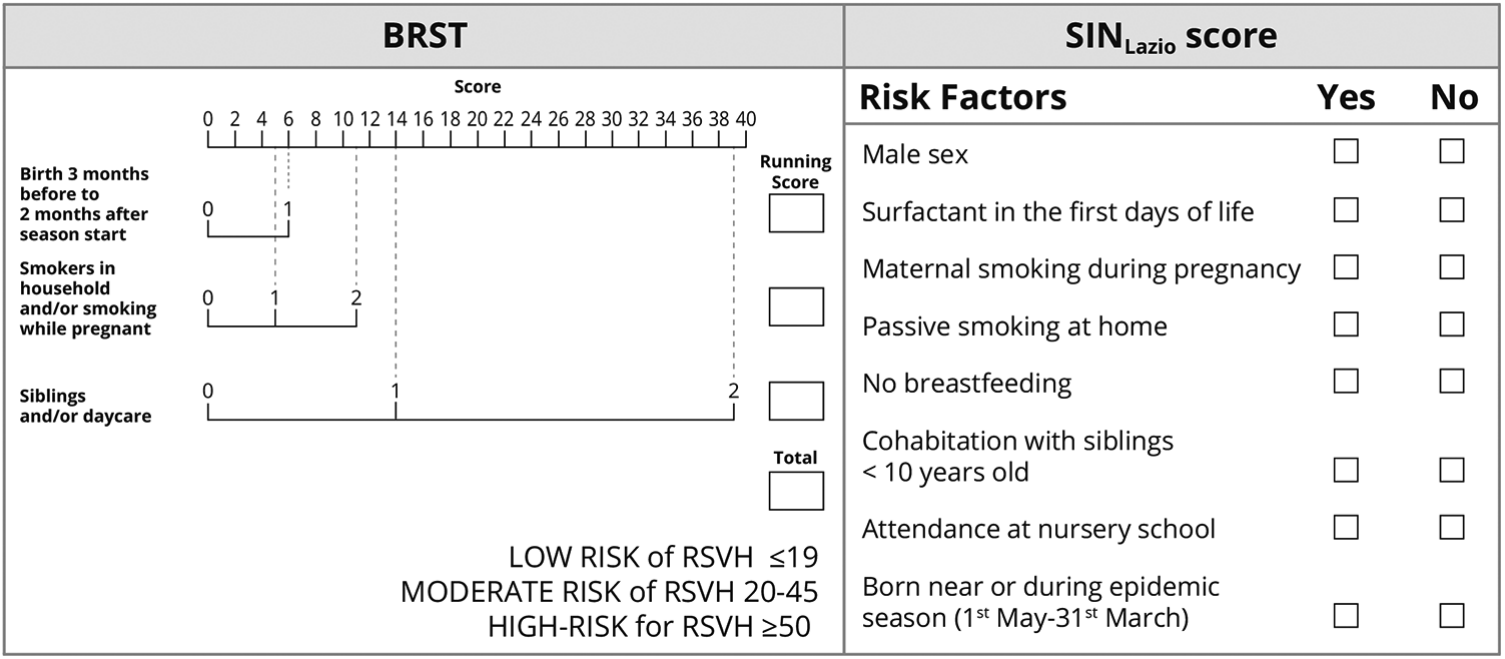
The risk factors taken into consideration in the Blanken Risk Scoring Tool (BRST) and in the SIN_Lazio_ score. In the BRST, each risk factor has a specific score. Adding up all the scores, an infant can be grouped into one of three identified categories (low, moderate, and high for RSV Hospitalization-RSVH-) (21). According to the SIN_Lazio_ score, infants with the presence of three or more risk factors need prophylaxis (23).

This is a retrospective analysis of the epidemiologic and clinical information of late preterm infants born between 2018 and 2020 and diagnosed with RSV infection at our site. Infants with the following criteria were included: (1) born at our unit (Neonatology-NICU Casilino General Hospital, Rome, Italy); (2) born during or 6 months before the two consecutive epidemic seasons 2018–2019 and 2019–2020; (3) moderate-to-late preterm born at 33–35^+6^. Infants with any of the following criteria were excluded: (1) BPD, (2) cystic fibrosis or congenital heart disease, (3) Down syndrome, (4) anatomic pulmonary abnormalities, and (5) neuromuscular disorders. Figure 1 illustrates a flowchart of participants in the study. The study participants were categorized according to both the SIN_Lazio_ and Blanken scores. The SIN_Lazio_ score is defined according to the previously described regional guidelines (23), while the BRST is calculated on the basis of the risk scoring tool previously described (21). Figure 2 illustrates the risk factors taken into consideration in both the BRST and the SIN_Lazio_ score. The BRST has a scale of 0–56 with defined cutoff values for low- (≤19), moderate- (20–45), and high-risk (≥50) infants. According to the BRST, infants with moderate risk are candidates for PLV. Infants received PLV 15 mg/kg in monthly dose courses over their first epidemic season (from November to March) according to existing protocols (SIN_Lazio_) and guidelines. Along with the assessment of risk factors, the following data were also collected for each patient when applicable: (1) onset of bronchiolitis; (2) age of onset; (3) hospitalization; (4) need of respiratory support; (5) doses of PLV before admission. Informed signed consent was obtained from the parents of all infants. This pilot retrospective analysis (ID number 150.22) was approved by the Ethics committee “Lazio 2” (Report number protocol. 0011318/2023 number. 0226066 of 21/11/2022).

### 2.1. Statistical analysis

Continuous variables were given as means with standard deviations (SD) and categorical variables as the number of subjects and percentage values. Based on the results from the derived contingency tables, sensitivity, specificity, and accuracy were calculated for the categorical SIN_Lazio_ score and BRST. The association among the categorical characteristics was assayed using Pearson's *χ*^2^ test (where appropriate, using Fisher's exact test). Moreover, univariate Penalised Logistic models were used to screen the effect of demographic–clinical characteristics on bronchiolitis, hospitalization, and hospital complications (the nasal cannulas, high flows, and O_2_ therapy, respectively). The odd ratios associated with outcomes were calculated with their 95% confidence interval for each factor from the Penalised Logistic model. The likelihood ratio test was used as the test of statistical significance. Due to the nature of the analysis, a pilot study in this case, the correction for multiple comparisons was not performed. Differences, with a *p*-value of less than 0.05, were considered significant, and data were acquired and analyzed in a R v4.0.3 software environment (26).

## 3. Results

### 3.1. Demographic and clinical data

The clinical data of 296 infants were retrospectively analyzed. The demographic and clinical characteristics of the study participants are illustrated in Table 1. Briefly, the mean GA and birth weight were 34.61 weeks (±1.2) and 2,230.45 grams (±410.56), respectively. A slightly greater proportion of infants were male [159/296 (53.7%)]. Approximately one-third of infants received PLV prophylaxis (94/296).

**Table 1:** Baseline demographic and clinical characteristics of study participants (*n* = 296).

Characteristic	Overall
Weeks of gestational age mean (SD)	34.61 (1.2)
Birth weight [g (mean, SD)]	2,230.45 (410.56)
**Gender**	
Male	159 (53.72%)
Female	137 (46.28%)
**PLV prophylaxis**	
No	202 (68.24%)
Yes	94 (31.76%)
**SIN_Lazio_ score**	
<3	173 (58.45%)
≥3	123 (41.55%)
**BRST**	
Low	248 (83.78%)
Moderate	48 (16.22%)
**Bronchiolitis**	
No	251 (84.8%)
Yes	45 (15.2%)
Age when diagnosed with bronchiolitis [months (mean, SD)]	5.33 (2.66)

The results are expressed as mean with standard deviation or as the number of subjects with percentage.

### 3.2. SIN_Lazio_ score vs. BRST: differences in selecting patients eligible for PLV prophylaxis

Based on the SIN_Lazio_ score, approximately 40% of all analyzed infants (123/296) would meet the criteria to be eligible for PLV prophylaxis. Twenty-nine patients eligible for prophylaxis according to the SIN score did not receive the drug for different reasons, including failure of their parents to give consent for drug administration and transfer outside of the Lazio region. In contrast to the SIN_Lazio_ score results, none of the analyzed infants would be considered at high risk (i.e., eligible for RSV prophylaxis based on international guidelines) according to the BRST, as most of them (173/296) displayed the lowest cutoff value (≤19), with only some having a moderate (20–45) cutoff value (48/296). A total of 45 (15.2%) bronchiolitis diagnoses were recorded on average at 5 months of age in the overall population.

Despite a significant association reported between the SIN_Lazio_ score and the BRST (*p* < 0.0001) (Table 2), almost 68.3% (84/123) of patients exhibiting ≥3 risk factors, and therefore eligible for RSV prophylaxis according to SIN_Lazio_ criteria, would not be receiving PLV if they were categorized on the basis of the BRST.

**Table 2:** Association between SIN_Lazio_ score and BRST among study participants (*n* = 296).

	SIN_Lazio_ score		*p*-value
	<3	≥3	
BRST			<0.0001
Low	164 (94.8%)	84 (68.29%)	
Moderate	9 (5.20%)	39 (31.71%)	
**Tot.**	173 (100%)	123 (100%)	

### 3.3. SIN_Lazio_ score and its association with bronchiolitis occurrence

To evaluate whether the SIN_Lazio_ criteria would also be informative in assessing the vulnerability of late preterm to RSV infection and therefore discriminating infants prone to bronchiolitis from healthy ones, we determined the accuracy, sensitivity, and specificity of the SIN_Lazio_ score. The SIN_Lazio_ score showed an accuracy rate of 61.81% [accuracy (95% CI): 61.81% (54.99%: 66.41%)] with a sensitivity and specificity of 61.35% and 57.78%, respectively. Of note, the BRST had an accuracy rate of 75.34% [accuracy (95% CI): 75.34% (70.02%: 80.14%)] with a sensitivity and specificity of 84.86% and 22.22%, respectively.

A univariate analysis was performed in both the entire population (*n* = 296, Table 3) and in infants with symptoms distinctive of bronchiolitis (*n* = 45, Table 4) to evaluate whether categorizing infants with the SIN_Lazio_ score would translate into differences in neonatal outcomes such as bronchiolitis and hospitalization incidence. In line with previously reported gender-related differences in RSV-related bronchiolitis (20), six out of ten (62.2%) males experienced bronchiolitis compared with less than four out of ten (37.7%) females who exhibited a 33% lower risk of having bronchiolitis [OR (95% CI) = 0.67 (0.35: 1.27)] than males. The univariate penalized logistic regression analysis (Table 3) demonstrated a significant association between the SIN_Lazio_ score and the bronchiolitis event (*p*-value = 0.0175). In detail, bronchiolitis occurrence in patients with a SIN_Lazio_ score ≥3 was approximately 2.2 times more likely than that in patients with a SIN_Lazio_ score <3 [OR (95% CI) = 2.15 (1.14: 4.12)]. Of note, bronchiolitis occurrence in patients with a moderate BRST was less than 2 times more likely than in patients with a low BRST [OR (95% CI) = 1.64 (0.73: 3.45)], and this difference was not significant (*p* = 0.2207). Among the infants experiencing bronchiolitis, almost 68.9% 31/45) did not receive PLV prophylaxis, and no differences were observed in the incidence of bronchiolitis between PLV-treated infants and those who did not receive RSV prophylaxis [OR (95% CI) = 0.98 (0.49: 1.90), *p* = 0.9548].

**Table 3:** Univariate analysis assessing the association between variable bronchiolitis, infants' chara cteristics, and eligibility criteria for RSV prophylaxis with PLV in the study population (*n* = 296).

Characteristics	Bronchiolitis		OR (95% CI)	*p*-value
	No [251 (84.8%)]	Yes [45 (15.2%)]		
Weeks of gestational age mean (SD)	34.65 (0.84)	34.73 (0.98)	1.11 (0.77: 1.63)	0.5801
Birth weight [g (mean, SD)]	2,243.71 (416.66)	2,156.44 (370.28)	1.00 (0.99: 1.01)	0.1859
Gender				0.2190
Male	131 (52.19%)	28 (62.22%)	1	** **
Female	120 (47.81%)	17 (37.78%)	0.67 (0.35: 1.27)	** **
BRST				0.2207
Low	213 (84.86%)	35 (77.78%)	1	** **
Moderate	38 (15.14%)	10 (22.22%)	1.64 (0.73: 3.45)	** **
SIN_Lazio_ score				0.0175
<3	154 (61.35%)	19 (42.22%)	1	** **
≥3	97 (38.65%)	26 (57.78%)	2.15 (1.14: 4.12)	** **
PLV prophylaxis				0.9548
No	171 (68.13%)	31 (68.89%)	1	** **
Yes	80 (31.87%)	14 (31.11%)	0.98 (0.49: 1.90)	** **

The results are expressed as mean with standard deviation or as the number of subjects with percentage; OR (95% CI): Odd Ratio with 95% Confidence Interval; *p*-value: Likelihood Ratio test *p*-value.

**Table 4:** Univariate analysis assessing the association between variable hospitalization, infants' characteristics, and eligibility criteria for RSV prophylaxis with PLV in infants diagnosed with bronchiolitis (*n* = 45).

Characteristics	Hospitalization (27)		*p*-value
	No [28 (62.22%)]	Yes [17 (37.78%)]	
Weeks of gestational age mean (SD)	34.66 (0.92)	34.83 (1.08)	0.6010
Birth weight [g (mean, SD)]	2,143.57 (320.3)	2,177.65 (450.72)	0.7613
Gender			0.3864
Male	16 (57.14%)	12 (70.59%)	** **
Female	12 (42.86%)	5 (29.41%)	** **
BRST			0.8391
Low	22 (78.57%)	13 (76.47%)	** **
Moderate	6 (21.43%)	4 (23.53%)	** **
SIN_Lazio_ score			0.0095
<3	16 (57.14%)	3 (17.65%)	** **
≥3	12 (42.86%)	14 (82.35%)	** **
PLV prophylaxis			0.0780
No	22 (78.57%)	9 (52.94%)	** **
Yes	6 (21.43%)	8 (47.06%)	** **
Pre-bronchiolitis PLV dose number^a^			0.0095
0	21 (80.77%)	7 (41.18%)	** **
1	4 (15.38%)	8 (47.06%)	** **
2	0 (0%)	2 (11.76%)	** **
3	1 (3.85%)	0 (0%)	** **

The results are expressed as mean with standard deviation or as the number of subjects with percentage; OR (95% CI): Odd Ratio with 95% Confidence Interval; *p*-value: Likelihood Ratio test *p*-value.

^a^ The data corresponding to the Pre-bronchiolitis PLV dosing course pertain to 43 out of 45 patients, because for the remaining two patients, the number of doses taken has not been recorded.

### 3.4. Ability of the SIN_Lazio_ score to predict the risk of hospitalization

The univariate penalized logistic regression analysis reported in Table 4 shows a statistically significant effect of the SIN_Lazio_ score on predicting the risk of hospitalization (*p* = 0.0095), with eight out of ten hospitalized patients having a SIN_Lazio_ score ≥3. Of note, the chances of being admitted to hospital for bronchiolitis in patients with a SIN score ≥3 were approximately 5.5 times more likely than in patients with a SIN_Lazio_ score <3 [OR (95% CI) = 5.47 (1.49: 24.92), data not provided]. More than one-third of patients with bronchiolitis (17/45, 37.7%) were hospitalized.

These infants required hospitalization for the following reasons: (i) difficulty in feeding (3 out of 17 patients, accounting for 17.6% of inpatients or 6.6% of patients with bronchiolitis); (ii) respiratory difficulties with the need for assistance. Specifically, four patients (23.5% of inpatients and 8.9% of those with bronchiolitis) needed O_2_-therapy; 10 patients (58.9% of inpatients and 22.2% of bronchiolitis) needed non-invasive ventilatory assistance (High-Flow Nasal Cannula, n-CPAP, or n-IPPV); no patients needed intubation (data not shown).

As summarized in Table 4, the 17 infants hospitalized for bronchiolitis would not be undergoing RSV prophylaxis on the basis of the analyzed scores. According to the BRST, > 70% of them (13/17) would not be eligible for PLV treatment. Conversely, most hospitalized patients displayed a SIN_Lazio_ score ≥3, thus suggesting that these infants could be the most vulnerable patients and may benefit the most from RSV prophylaxis. Among the infants diagnosed with bronchiolitis (Table 3), only 14 out of 45 (31.1%) received PLV prophylaxis; in detail, among the infants hospitalized, namely 17 out of 45, only eight were administered PLV prophylaxis (Table 4). As indicated in Table 4, most of the hospitalized infants [15/17 (88.2%)] have received up to one dose of PLV, thus not availing the benefit of a complete treatment course. The burden of bronchiolitis is relevant for the NICU, thus requiring an increased need for respiratory support. Therefore, minimizing this need may be of relevance. Of note, the risk of requiring a nasal cannula was reported being 91% lower in patients who received PLV than in those not receiving any PLV prophylaxis [OR (95% CI) = 0.09 (0.01: 0.68), *p* = 0.0174, data not shown]. Among admitted infants, nine had an RSV-positive swab, and all nine did not receive PLV. When evaluating risk factors, the analysis revealed that only two had a moderate BRST. In line with the SIN_Lazio_ score, six infants had a score ≥3, but they did not receive PLV either because of transfer to another country or because of the refusal of their parents.

## 4. Discussion

It has been well documented that prematurity alone is a significant risk factor for RSV-related hospitalization and severe disease (2, 9, 28, 29), with mid-to-late preterm (infants born between 33 and 35 wGA) being at a significant risk for severe RSV infections. Mounting evidence supports the respiratory vulnerability of moderate-to-late preterm infants that stems from both the early interruption of pulmonary development and the physiologic immaturity of the immune system (2, 30). In addition, infants born preterm have lower levels of maternal antibody that can provide protection against respiratory pathogens (31). Therefore, such population should be targeted by RSV prophylaxis instead of being denied access to PLV treatment, as it is considered low risk in several guidelines (15). As underlined by Priante et al., while it is hard to establish a definite gestational age “threshold” distinguishing between high-risk and low-risk infants, current evidence indicates that preterm infants born at ≤35 wGA are all at risk for severe RSV-related disease than term-born infants, particularly in the early months of life (32). In line with this, previous Italian data support the use of PLV prophylaxis for otherwise healthy preterm (29–35 wGA) infants aged ≤6 months at the beginning of the RSV season (2). Moreover, extending PLV prophylaxis to 29–32 wGA infants appeared to be a cost-effective strategy (33) along with a calculated cost per quality-adjusted life years (QALY) of 14,937.32 € when accomplished in premature infants born between 33 and 35 wGA (34). However, whether PLV prophylaxis could be provided for preterm infants born between 32 and 35 wGA without chronic lung disease and hemodynamically significant congenital heart disease remains a matter of high debate (35).

To aid the targeting of prophylaxis for infants born between 33 and 35 wGA at risk for RSV hospitalization, the use of a risk factor model and scoring tool can be helpful in promoting decision-making for clinicians and policymakers. Multifactorial risk scores to identify high-risk moderate/late-preterm infants have been suggested (36, 37) along with a risk scoring tool to predict hospitalization in moderate-late preterm infants (21). In our retrospective analysis, we evaluated whether the adoption of less or more restrictive prophylaxis eligibility criteria (namely, then SIN_Lazio_ score and BRST) translated into differences in neonatal outcomes (e.g., bronchiolitis and incidence of hospitalization) in a cohort of vulnerable moderate-to-late preterm infants born during two consecutive seasons (2018–2019 and 2019–2020). The SIN_Lazio_ score allowed us to identify a significant proportion of infants to be considered for prophylaxis, precisely 123 infants with a SIN_Lazio_ score ≥3. Interestingly, in the same overall population, according to the BRST as indicated in a recent consensus (20), none of the infants should be considered at risk and subsequently eligible for RSV prophylaxis. Therefore, the adoption of a risk scoring tool based on three risk variables [e.g., birth between 3 months before and 2 months after season start date, smokers in the household and/or maternal smoking while pregnant, and siblings (excluding multiples) and/or (planned) daycare attendance] would deny vulnerable infants the opportunity of prophylaxis. Moreover, the observation that those infants with a SIN_Lazio_ score ≥3 also had a higher risk of bronchiolitis (2.2-fold increase) and of hospitalization (5.5-fold increase) further supports the eligibility of these children for RSV prophylaxis. While a direct comparison between SIN_Lazio_ and the BRST is not possible as their scoring system is based on similar but not identical parameters, our preliminary data seem to suggest that the adoption of a less restrictive eligibility criteria for PLV prophylaxis may help in identifying infants who could benefit the most from prophylaxis, thereby minimizing the rate of RSV-associated hospitalization and ultimately its long-term clinical sequelae. In our retrospectively analyzed population, approximately 37.7% of infants diagnosed with bronchiolitis (17/45) were hospitalized, with most of them classified as eligible for RSV prophylaxis according to the SIN_Lazio_ score, but not according to the BRST, for which approximately 70% (13/17) of them would be neither at risk for hospitalization nor eligible for PLV treatment.

PLV is a primary prevention strategy for RSV infection worldwide (38). Although the data suggest that PLV treatment is associated with a lower incidence of bronchiolitis and use of hospital-related healthcare resources, in our analysis, the results are not statistically significant due to the small sample size and the observation that most infants with bronchiolitis did not receive the approved PLV treatment regimen (e.g., five injections during the RSV season in an interval of 4 weeks, up to 5 times). Most infants received not more than one dose and a few of them up to 3 doses. This apparent lower RSV protection observed in PLV-treated infants may stem from the observation that, especially between the first and the second injection, the interval of 4 weeks should be strictly adhered to prevent breakthrough infections (an infection occurring despite prophylaxis between the first and the second injection). Accordingly, it has been suggested that the current dose-interval scheme of PLV (administration every 30 days, for 5 months during the epidemic season) may not deliver full protection throughout the entire 1-month interval between doses (39, 40). In addition, a reduced number of PLV doses was reported being unable to protect against severe RSV disease over a typical 5-month RSV season (39–42). Finally, although PLV treatment is recommended to be started on November 1 in our region (23), we could not retrieve the start date for all the PLV-treated infants, and it is likely that not all infants are appropriately receiving PLV treatment according to an optimal timing. It has been well-documented that the timing of the treatment can affect clinical outcomes significantly (43, 44). As a result, starting too early or too late may leave patients vulnerable for a part of the season. Accordingly, delays in administering PLV at the beginning of the season were found to increase the rate of RSV infection–related hospitalization (45). Overall, infants who qualify for PLV treatment should receive the treatment as early as possible and according to a complete 5-dose course protocol.

We acknowledge that the present retrospective analysis has several limitations. Particularly, a low number of patients with RSV infection was observed because pharyngeal swabbing is performed exclusively for patients requiring hospitalization (in our case, 17 out of 45 infants). Of these 17 patients hospitalized with bronchiolitis and who underwent a swab analysis for RSV, 9 were positive for the virus of our interest. It was not possible to assess whether hospitalized patients (both those with negative and RSV-positive swabs) were positive for other respiratory viruses, because in the analyzed two seasons, it was not planned to routinely evaluate infection with other respiratory viruses in infants. In addition, this low number of RSV-positive patients stems from the small simple size, because the data were collected only from a single third-level hospital. Finally, the estimated OR with 95% confidence intervals computed for assessing the effects of the demographic–clinical factors on hospitalization and hospital-related complications could be influenced by the small sample size.

## 5. Conclusion

Our preliminary findings support the need for targeting late preterm infants for RSV prophylaxis and, pending further studies and analyses in a larger preterm infant population, call for an appraisal of the current eligibility criteria for PLV treatment to help in the better management of the consequences of RSV infection in vulnerable infants. In addition, our work confirms the importance of acknowledging the eligibility for RSV prophylaxis for moderate-to-late term infants because of their respiratory vulnerability and physiologic immaturity of their immune system that place them at a higher risk of experiencing bronchiolitis. Therefore, adopting less restrictive criteria for eligibility may ensure a comprehensive prophylaxis of the eligible subjects, thus sparing them from avoidable short- and long-term consequences of RSV infection. The evolution in knowledge about the immune response against RSV, as well as the increasing identification of RSV disease burden, has led to an important increase in the number of promising candidates for active and passive immunization. Presently, 3 monoclonal antibodies and 17 active immunization candidates are under development in phase 1 to 3 clinical studies (27). With regard to the first group, Nirsevimab is a monoclonal antibody with a prolonged half-life with approval for clinical use in infants (46, 47). In the coming years, a strategy for the protection of infants and preschoolers could be achieved with a combination of different approaches such as passive immunization, use of monoclonal antibodies, and maternal vaccination during pregnancy.

## Data Availability

The raw data supporting the conclusions of this article will be made available by the authors without undue reservation.
